# The first assimilation of Akatsuki single-layer winds and its validation with Venusian atmospheric waves excited by solar heating

**DOI:** 10.1038/s41598-022-18634-6

**Published:** 2022-08-26

**Authors:** Yukiko Fujisawa, Shin-ya Murakami, Norihiko Sugimoto, Masahiro Takagi, Takeshi Imamura, Takeshi Horinouchi, George L. Hashimoto, Masaki Ishiwatari, Takeshi Enomoto, Takemasa Miyoshi, Hiroki Kashimura, Yoshi-Yuki Hayashi

**Affiliations:** 1 Research and Education Center for Natural Sciences, Keio University, 4-1-1, Hiyoshi, Kohoku-ku, Yokohama, Kanagawa 223-8251 Japan; 2 Institute of Space and Astronautical Science, Japan Aerospace Exploration Agency, 3-1-1 Yoshinodai, Chuo-ku, Sagamihara, Kanagawa 252-5210 Japan; 3 Department of Physics, Keio University, 4-1-1, Hiyoshi, Kohoku-ku, Yokohama, Kanagawa 223-8251 Japan; 4 Department of Astrophysics and Atmospheric Science, Kyoto Sangyo University, Kamigamo Motoyama, Kita-ku, Kyoto, 603-8555 Japan; 5 Graduate School of Frontier Sciences, The University of Tokyo, 5-1-5 Kashiwanoha, Kashiwa, Chiba 277-8561 Japan; 6 Faculty of Environmental Earth Science, Hokkaido University, Kita 10, Nishi 5, Kita-ku, Sapporo, Hokkaido 060-0810 Japan; 7 Department of Earth Sciences, Okayama University, 3-1-1, Tsushimanaka, Kita-ku, Okayama, 700-8530 Japan; 8 Department of Cosmosciences, Hokkaido University, Kita 10, Nishi 8, Kita-ku, Sapporo, Hokkaido 060-0810 Japan; 9 Disaster Prevention Research Institute, Kyoto University, Gokasho, Uji, Kyoto 611-0011 Japan; 10 Application Laboratory, Japan Agency for Marine-Earth Science and Technology, 3173-25, Showamachi, Kanazawa-ku, Yokohama, Kanagawa 236-0001 Japan; 11 RIKEN Research Center for Computer Science (R-CCS), 7-1-26, Minatojima-minami-machi, Chuo-ku, Kobe, Hyogo 650-0047 Japan; 12 Center for Planetary Science, Kobe University, 7-1-48, Minatojima-Minamimachi, Chuo-ku, Kobe, Hyogo 650-0047 Japan; 13 Department of Planetology, Kobe University, 1-1, Rokkodai, Nada-ku, Hyogo, Kobe 657-8501 Japan

**Keywords:** Planetary science, Atmospheric dynamics

## Abstract

The planetary missions including the Venus Climate Orbiter ‘Akatsuki’ provide new information on various atmospheric phenomena. Nevertheless, it is difficult to elucidate their three-dimensional structures globally and continuously only from observations because satellite observations are considerably limited in time and space. We constructed the first ‘objective analysis’ of Venus’ atmosphere by assimilating cloud-top horizontal winds on the dayside from the equator to mid-latitudes, which is frequently obtained from Akatsuki's Ultraviolet Imager (UVI). The three-dimensional structures of thermal tides, found recently to play a crucial role in maintaining the super rotation, are greatly improved by the data assimilation. This result is confirmed by comparison with Akatsuki's temperature observations. The momentum transport caused by the thermal tides and other disturbances are also modified by the wind assimilation and agrees well with those estimated from the UVI observations. The assimilated dataset is reliable and will be open to the public along with the Akatsuki observations for further investigation of Venus’ atmospheric phenomena.

## Introduction

The Venus orbiter Akatsuki has observed Venus with several wavelengths since December 2015^1,2^. Its data provide new information on various atmospheric phenomena, including the superrotation^3–5^, streak structures^6,7^, bow-shaped patterns^8,9^, thermal tides^10–14^, planetary-scale short-period waves, such as Rossby waves^15–18^ and gravity waves^19–24^. These phenomena are crucial to understanding the atmospheric general circulation of Venus^25^. Nevertheless, elucidating their three-dimensional structures globally and continuously only from observations is difficult because satellite observations are limited in time and space. To obtain spatiotemporally homogeneous three-dimensional data, called objective analysis (e.g.^26^), assimilation methods, which incorporate observations into a numerical model, have been developed for the Earth atmosphere to perform numerical weather forecast. The objective analysis has also been developed for the Martian atmosphere (e.g.^27^), and its products are widely used in planetary science studies. Nevertheless, for the atmosphere of Venus, the application of data assimilation remains at the initial stage compared to the Earth and Martian atmosphere cases, because of limited observational coverage and premature atmospheric models.

Recently, based on a data assimilation scheme named local ensemble transform Kalman filter (LETKF)^28,29^ and the general circulation model (GCM) named atmospheric GCM for the Earth Simulator for Venus (AFES-Venus)^30^, the first data assimilation system for the atmosphere of Venus, named AFES-LETKF data assimilation system for Venus (ALEDAS-V)^31^, was developed. Using ALEDAS-V, Sugimoto et al.^32^ succeeded in demonstrating improvement of the spatial structure of the thermal tides by assimilating cloud-top horizontal winds derived from ultraviolet images obtained by the Venus Monitoring Camera (VMC) onboard ESA’s Venus Express.

The thermal tides are planetary-scale atmospheric waves excited by the diurnal component of solar heating. Because approximately 60% of the solar flux incident on Venus is absorbed in the cloud layer (45–70 km)^33^, the thermal tides are strongly excited there and are considered to play crucial roles in the atmosphere of Venus general circulation (e.g.^34–39^). The ALEDAS-V study^32^ demonstrated that even highly limited wind data can profoundly modify the simulated Venus atmosphere through data assimilation. The AFES-Venus version they applied, which is also used in this study, is a simple GCM without sophisticated cloud and radiation processes, but the atmosphere is driven by a prescribed profile of solar heating and infrared cooling.

The VMC used in Sugimoto et al.^32^ mainly observes the southern hemisphere once a day, while the Akatsuki UVI^40^, which is used in the present study, specifically observes equatorial latitudes at least once in every 6 h for the period we used. The reliability of assimilation by the use of Akatsuki UVI data is expected to be better than Sugimoto et al.^32^ especially for the northern hemisphere where frequent observation covers a wider area from the equator to mid-latitudes. The good coverage of equatorial latitudes provided by Akatsuki UVI data would be particularly effective in reproducing thermal tides and produce a consistent estimate of momentum transport caused by thermal tides. In this study, we employ ALEDAS-V to assimilate horizontal winds derived from the Akatsuki UVI images to produce the first objective analysis of the atmosphere of Venus and validated against a free run (FR) and the Akatsuki UVI, Akatsuki Longwave Infrared Camera (LIR), and Radio Occultation (radio science, RS)^5,14,41^ observations with a focus on the thermal tides. See Methods for details.

## Results

### Structure of horizontal winds associated with thermal tides

**Figure 1:**
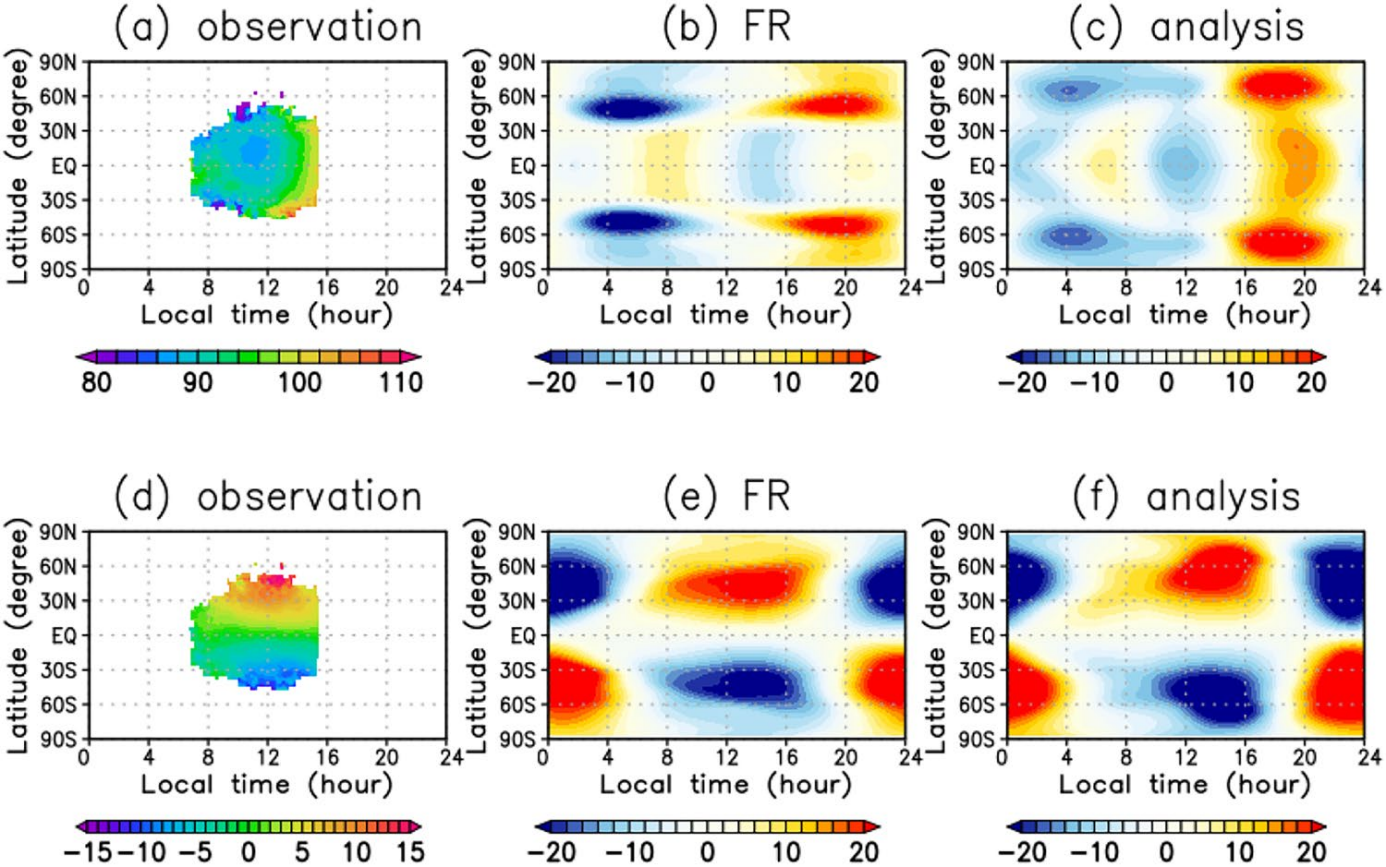
Local time and latitudinal distributions of (**a–c**) zonal winds (m s^−1^) and (**d–f**) meridional winds (m s^−1^) associated with thermal tides at the cloud-top (~ 70 km) level: (**a**, **d**) observation, (**b**, **e**) FR and (**c**, **f**) analysis. These distributions are averaged for the two Earth months from 1 October to 30 November 2018 in a reference frame moving with the Sun to extract the structures associated with thermal tides. Deviations from the zonal means of zonal and meridional winds are shown in (**b**, **c**) and (**e**, **f**), respectively.

We assimilate cloud-top zonal and meridional winds obtained by tracking small-scale clouds using UVI data^42^ from 1 September to 31 December in 2018 and analyse the assimilated data from 1 October to 30 November 2018, in which values of the root-mean-square-deviation (RMSD) from FR are stable (see Methods). Figure 1a and d show the zonal and meridional winds obtained from the Akatsuki UVI images, respectively. They are averaged for the 2 months in a reference frame moving with the Sun and hence mainly include the zonal-mean and thermal-tide components. Note that the direction of the planetary rotation is set to be eastward (negative) following the convention of geophysical fluid dynamics, and consistently the zonal mean flow and the movement of the Sun are all opposite to the real Venus. This is the setting convenient for those familiar with the Earth’s atmospheric dynamics. The magnitude of the zonal wind has a local minimum near the equator around 11 LT. This minimum is located around 20°N and the distribution of the zonal-mean wind is not slightly symmetric about the equator (Fig. 1a). The meridional winds are poleward, and their magnitude increases with latitude and are longitudinally centred around noon (Fig. 1d)^12,37,43–45^. Note that the wind observations cover only the dayside of about 7–15 LT equatorward of 50° latitudes.

Figure 1b and e show the tidal winds defined as deviations from zonal means at 70 km altitude in the FR. The zonal wind indicates that the diurnal (zonal wavenumber 1) and semi-diurnal (zonal wavenumber 2) tides are predominant^46^ at latitudes poleward and equatorward of 30°, respectively (Fig. 1b). The phase difference in the equatorial region between the UVI observations and the FR is prominent. The zonal wind deviation of FR disappears around noon, and it has a local minimum at 14–15 LT, which is delayed by approximately 2 h in comparison with that of the UVI observations (Fig. 1a and b). The meridional winds, which are dominated by the diurnal tide (Fig. 1e), are poleward and equatorward in the dayside and nightside, respectively. This distribution is consistent with the Akatsuki observations (Fig. 1d)^47^, suggesting that the phase of the diurnal tide is well reproduced in the FR.

Figure 1c and f show the tidal winds in the analysis (obtained by assimilating the UVI horizontal winds). Now, the zonal winds in the equatorial region have a local minimum around 11 LT (Fig. 1c). Thus, the assimilation changed the semi-diurnal tide to approach the observations (Fig. 1a), as expected. Moreover, the local minimum is slightly shifted to the north, with the north–south asymmetry consistent with the observations. Interestingly, the horizontal wind field is significantly modified, even on the nightside, although the observed winds are available only in the dayside. Furthermore, the local maximum (minimum) around 40° latitude in the FR are shifted to around 70° latitude in the analysis, and their structures exhibit slight north–south symmetry (Fig. 1c). This result suggests that the winds assimilation limited to low latitudes on the dayside strongly affects global atmospheric motions in the GCM, and the global circulation in the GCM could be improved by assimilating spatially limited data. Additionally, the amplitudes of the diurnal and semi-diurnal tides increased. In the FR, the amplitudes are approximately 10 m s^−1^ at the equator, whereas they are approximately 15 m s^−1^ in the analysis. Their amplitudes in the FR are underestimated compared with the observations. The meridional winds have the maximum (or minimum) at around 40° latitude in the FR, although it shows a spread towards higher latitudes in the analysis (Fig. 1f). However, compared with the large modifications in the zonal winds, the assimilation only slightly changed the meridional winds of the thermal tides. This may be due to the similarity of observations and FRs at lower latitudes, while there are fewer assimilating observations at around 50° latitude (see Methods).

### Structure of temperature associated with thermal tides

**Figure 2:**
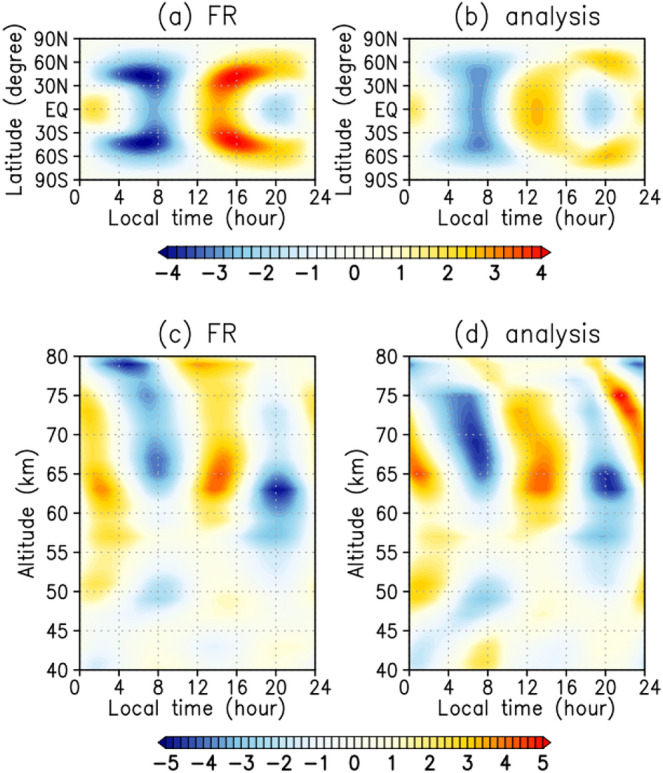
(**a**, **b**) Local time and latitudinal distributions at the cloud-top altitudes and (**c**, **d**) local time and vertical distributions at the equator of temperature (K) associated with the thermal tides: (**a**, **c**) FR and (**b**, **d**) analysis. (**a**, **b**) Vertically averaged distributions with the weighting function^48^ are plotted to compare with the results of LIR observations. (**c**, **d**) Amplitudes are scaled with the square root of the density at each altitude divided by the density at 70 km altitude for visibility. Data are averaged for the two Earth months at each altitude in a reference frame moving with the Sun to extract the structures associated with thermal tides. Deviations from the zonal means of temperature are shown.

Figure 2a and b show the tidal components of the temperature obtained in the FR and analysis, respectively, which are vertically averaged with a weighting function of LIR^48^ to compare with the LIR observations^14^. As for the zonal winds, the FR temperature is dominated by the diurnal and semi-diurnal tides at latitudes poleward and equatorward of 30°, respectively (Fig. 2a). The temperature deviation vanishes around 11 LT and has a local maximum at 14–15 LT in the equatorial region. This distribution is not consistent with the LIR observations^14^, in which the temperature distribution has two maxima in the morning (9 LT) and evening (20 LT) regions and two minima at approximately 2 and 15 LT. Namely, the phase distribution of the semi-diurnal tide in the FR is zonally shifted by 6 h from the observations. By contrast, the temperature in the analysis (Fig. 2b) has a local maximum at around 13 LT, indicating that the temperature field also improves by the data assimilation, although the temperature observations were not assimilated.

We also compare the temperature fields with those obtained by RS^11,41^. Figure 2c and d show the equatorial vertical cross-sections of the tidal components of temperature in the FR and analysis, respectively, which are scaled with the square root of density at each altitude divided by the density at 70 km altitude. In both the FR and analysis, the semi-diurnal tide is dominant, which is consistent with the RS results^41^. The phase structures of the thermal tides are similar between the two cases at around 65 km, where the strong solar heating creates vertical maxima in the normalised temperature fields. In the FR, the tidal temperature field has an upright structure, but it is tilted in the analysis. This titled feature is more consistent with Akatsuki RS results^41^. These results indicate that the three-dimensional structure of the semi-diurnal tide has been improved by only assimilating the horizontal winds at the dayside cloud-top. The amplitude of the tidal temperature field is also enhanced by the horizontal wind assimilation in altitudes of 70–75 km. The magnitude in the analysis at 70 km is approximately 5 K, which is approximately 1 K larger than that in the FR. In contrast, at 80 km, the amplitude of thermal tides in the analysis is smaller than that in the FR. This result indicates that the thermal tides are more strongly attenuated along their vertical propagation in the real Venus atmosphere than in the GCM.

### Angular momentum transport associated with thermal tides

**Figure 3:**
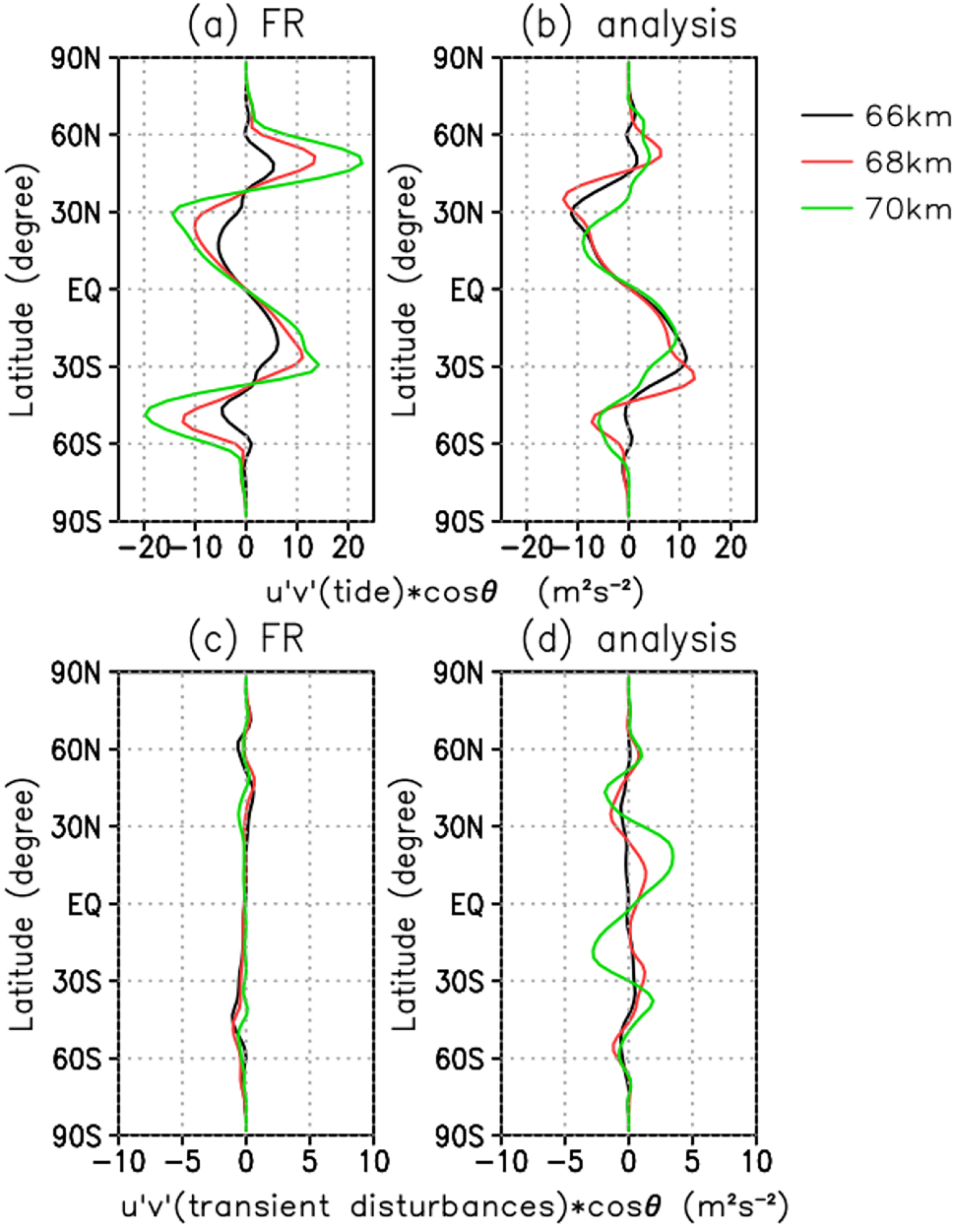
Latitudinal distributions of zonal-mean meridional angular momentum fluxes (m^2^ s^−2^) due to (**a**, **b**) thermal tides and (**c**, **d**) transient disturbances near the cloud-top level averaged for the two Earth months: (**a**, **c**) FR and (**b**, **d**) analysis: The black, red and green lines are shown at 66, 68 and 70 km levels, respectively. Note that when the flux is positive, the angular momentum is transported northward (see “Methods”).

Figure 3 shows the meridional angular momentum fluxes near the cloud top (66–70 km) averaged over two Earth months (see Methods). For the angular momentum fluxes associated with the thermal tides (Fig. 3a and b), both FR and analysis fluxes are negative (positive) in the northern (southern) hemisphere from the equator to approximately 40° N (S) latitudes, indicating the equatorward transport of prograde angular momentum in both hemispheres. The locations of negative (positive) peaks depend on altitude but are localised in latitudes between 20 and 40° N (S). These results agree with those obtained from the UVI observations, where the thermal tides induce equatorward angular momentum fluxes to contribute to the acceleration of super-rotation^5^. In contrast, at latitudes between 40 and 60° N (S) in the FR, the fluxes are largely positive (negative) in the northern (southern) hemisphere. In the analysis, the momentum fluxes at those latitudes significantly decrease, which is closer to the UVI observations^5^.

The angular momentum fluxes for transients, which are defined as waves and/or disturbances other than the thermal tides, are also estimated for both FR and analysis (Fig. 3c and d). The observational study indicates that short-period disturbances contribute to the deceleration of super-rotation^5^. The amplitudes of the FR fluxes are much smaller than those in UVI observations, while those of the analysis fluxes are somewhat increased, indicating that the transients are also improved by analysis. This result is surprising because the non-tidal transient disturbances are intermittent and less constrained by the observation than the thermal tides.

### Zonal-mean zonal wind and temperature

**Figure 4:**
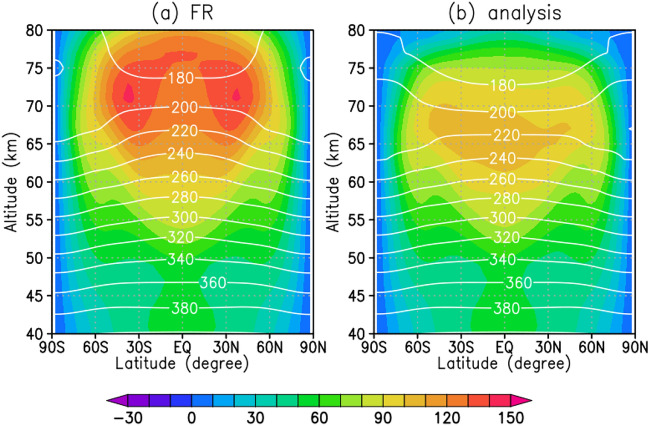
Latitudinal and vertical distributions of temporal and zonal-mean zonal wind (colour, m s^−1^) and temperature (contour, K) averaged in two Earth months: (**a**) FR and (**b**) analysis.

Figure 4 shows latitudinal and vertical cross-sections of the zonal-mean zonal wind and temperature fields for the FR and analysis averaged for 2 months. They are largely altered by the data assimilation; the zonal-mean zonal wind of the FR has a maximum of 150 m s^−1^ at 70–75 km altitudes, whereas it has a maximum of 120 m s^−1^ at 65–70 km altitudes in the analysis. Because the UVI observations show that the maximum of the zonal wind was at 80–110 m s^−1^ in the low latitudes on the dayside (Fig. 1a), assimilation of those zonal wind can fairly improve the zonal-mean zonal wind. Correspondingly, because of the thermal wind relation, the meridional temperature gradient reduces in the analysis, especially in high latitudes at 65–75 km altitudes. Note that the zonal wind and temperature fields change over wide altitudes beyond 70 km where the observational data are given. On the equator at 80 km altitude, the zonal wind is strongly decelerated to 30 m s^−1^ in the analysis from 120 m s^−1^ in the FR at the equator. This deceleration could be explained by the negative zonal momentum deposit due to the attenuation of the enhanced thermal tides in the analysis. This result strongly indicates that the thermal tides have a large impact on the general circulation of the atmosphere of Venus.

### Discussions and conclusions

In this study, we produce the objective analysis of the atmosphere of Venus using ALEDAS-V by assimilating horizontal winds derived from Akatsuki UVI images, the first data assimilation product using the Akatsuki observations. Comparing the LIR and UVI observations with the analysis, the semi-diurnal tides in the equatorial region were particularly improved. Comparing the RS observation and the analysis, the tilts of the vertical structures were mainly improved. Although the assimilated observations are limited to the cloud-top horizontal winds in the dayside, the analysis widely modifies not only the horizontal wind fields but also the temperature field over a wide vertical range. These results strongly indicate that the improvements of the thermal tides are crucially important to reproduce the realistic general circulation in the Venus atmosphere because it is strongly affected by angular momentum and heat transports caused by the thermal tides, which are also modified by the data assimilation.

In the previous test study^32^ of data assimilation with the VMC, the data coverage was only the southern hemisphere once a day. It was shown that, in the southern hemisphere, amplitude peaks of zonal winds at the altitude of 70 km are located at around 40°S in the FR and around 70°S in the assimilation, respectively, while in the northern hemisphere, the zonal wind patterns in the mid- to high-latitude regions are similar in the FR and the assimilation. Thus, the assimilation results obtained by the previous study^32^ appear to have a north–south asymmetry from mid- to high latitudes, indicating low confidence in the results of the northern hemisphere.

The Akatsuki wind data used in the present study covers up to 50°N(S) latitudes on both hemispheres, with at least one observation every 6 h in the observation period we used. As a result, the analysis shows that the mid-latitude amplitude peaks are shifted towards higher latitudes and their north–south symmetry is conspicuous and hence considered to be reliable (Fig. 1c). Furthermore, in the Akatsuki observations, the local minimum of zonal wind is at about 20°N around 11 LT (Fig. 1a), indicating the north–south asymmetry. The analysis shows that the minimum of the zonal wind is slightly shifted to the north side of the equatorial region, reflecting the north–south asymmetry in the observations. The analysis obtained in the present study reproduces the observations more correctly.

While the analysis for the zonal winds is closer to the observations than in the former test study^32^, the analysis for the temperature is not sufficiently improved. Comparing the analysis (Fig. 2b) with the LIR observation^14^, the temperature profile has a maximum at 9 LT in the LIR observation and 13 LT in the analysis, with a difference of 4 LT between them. Comparing the analysis (Fig. 2d) with the RS observations, the observed values are ~ 6 and ~ 5.7 K at 70 and 79 km respectively in the RS observations, but the analysis values are ~ 5 and ~ 0 K at 70 and 80 km, respectively, indicating that the amplitudes of the thermal tides are decayed.

In order to solve these discrepancies in the future, for the data assimilation techniques, further improvements should be made, for example, by adjusting the parameters of the LETKF and improving quality control of the observations. The LIR and RS data are used for validation in this study, but they can also be assimilated to provide and additional observational constraints. In this study, we showed that our simple GCM, AFES-Venus, does not correctly reproduce the observed thermal tides without data assimilation. It is important to improve the GCM to correctly reproduce the thermal tides and the zonal-mean zonal wind. We are now trying to change the heating profile and the radiative scheme to improve the tides in FR.

In this study, we mainly focused on the thermal tide in the analysis for just one period. In the previous data assimilation test studies, it has been reported that the cold collar^49^ and planetary-scale Kelvin waves^50,51^ could be reproduced with sufficiently frequent observations. We are now planning to produce analyses with a number of observation periods, with which such kind of short period disturbances can be reproduced and investigated widely in the Venus research community. The assimilated data would be also helpful to elucidate the reason for long term variation of the super-rotation through momentum transports of long and/or short period disturbances^52^.

## Methods

### Numerical model and experimental settings

ALEDAS-V uses AFES-Venus and LETKF for ensemble forecasts and data assimilation, respectively. AFES-Venus solves primitive equations in sigma coordinate on a sphere, and physical parameters are chosen for the atmosphere of Venus^30^. The horizontal grids are 128 × 64 at each level (the triangular truncation number for spherical harmonics is 42). The vertical layers are 60 with a constant interval of 2 km from the flat ground to 120 km. The eddy diffusion is implemented both vertically and horizontally, with a constant coefficient of 0.15 m s^−2^ and the second-order hyper-viscosity with 0.1 Earth days for a damping time of the maximum wavenumber, respectively. A convective adjustment scheme is also introduced. The surface friction is represented by Rayleigh friction with 0.5 Earth days for a damping time. A sponge layer is introduced above 80 km only for the eddy components, and its damping times gradually decrease with altitude. The solar heating is based on Tomasko et al.^33^. A Newtonian cooling scheme based on Crisp^53^ is applied for the infrared radiative process. Horizontally uniform temperature based on the Venus International Reference Atmosphere^54^ is set to a relaxing field for Newtonian cooling. As an initial state, an idealised super-rotation in a solid-body rotation is set. The zonal wind increases linearly with altitude. At 70 km, the equator’s maximum velocity is 100 m s^−1^. Temperature is in gradient wind balance with super-rotation. The direction of the planetary rotation is set to be eastward following the conventional direction of rotation used in geophysical fluid dynamics, and consistently the zonal mean flow and the movement of the Sun are all opposite to the real Venus. This is the setting convenient for those familiar with the Earth’s atmospheric dynamics. Non-linear numerical simulations about four Earth years are performed for spin up. Eight-hourly outputs are used for the initial conditions of each 31-member ensemble forecast.

The LETKF is based on an ensemble Kalman filter (EnKF)^28,29^. The ensemble members are 31. The horizontal and vertical localisation parameters are 400 km and log *P* = 0.4 (where P is pressure), respectively. The inflation with 10% is set. The six-hourly time interval is set for the four-dimensional LETKF; then, seven-hourly time slots are used at each analysis. Thus, if the observations exist, they are assimilated every hour. We have also prepared the case without data assimilation named FR (free run). Details are described in the previous works^30–32,55^.

### Observation data

The observation data used in this study are the zonal and meridional winds obtained using a cloud tracking technique from images taken by UVI with a 365 nm filter of Akatsuki from September to December 2018^4,5,42,56,57^. This period includes the intensive observation period in November 2018 and provides relatively continuous observations over several months. The precision screening parameter ε in Ikegawa and Horinouchi^56^ is chosen as 10, and this parameter is a threshold value for an uncertainty measure of peak in the cross-correlation surface; greater value accepts broader tails of the peak. The parameter alpha in Horinouchi et al.^57^ is set to 2, and this parameter is a compatibility parameter used by the relaxation labeling method. These horizontal winds are obtained only in the dayside region from 60°S to 60°N near the cloud tops (~ 70 km).

**Figure 5:**
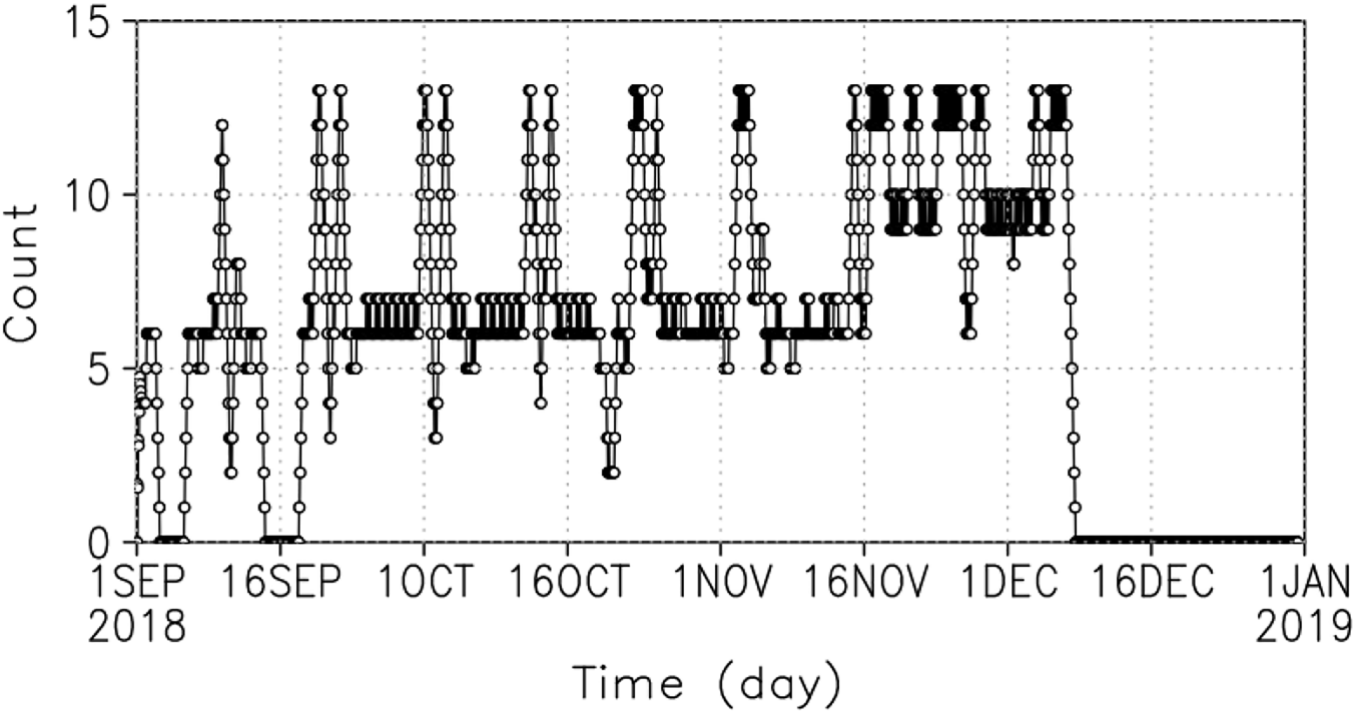
Time variation of observation frequency. Each point represents the sum of the observations for the preceding and following 12 h.

**Figure 6:**
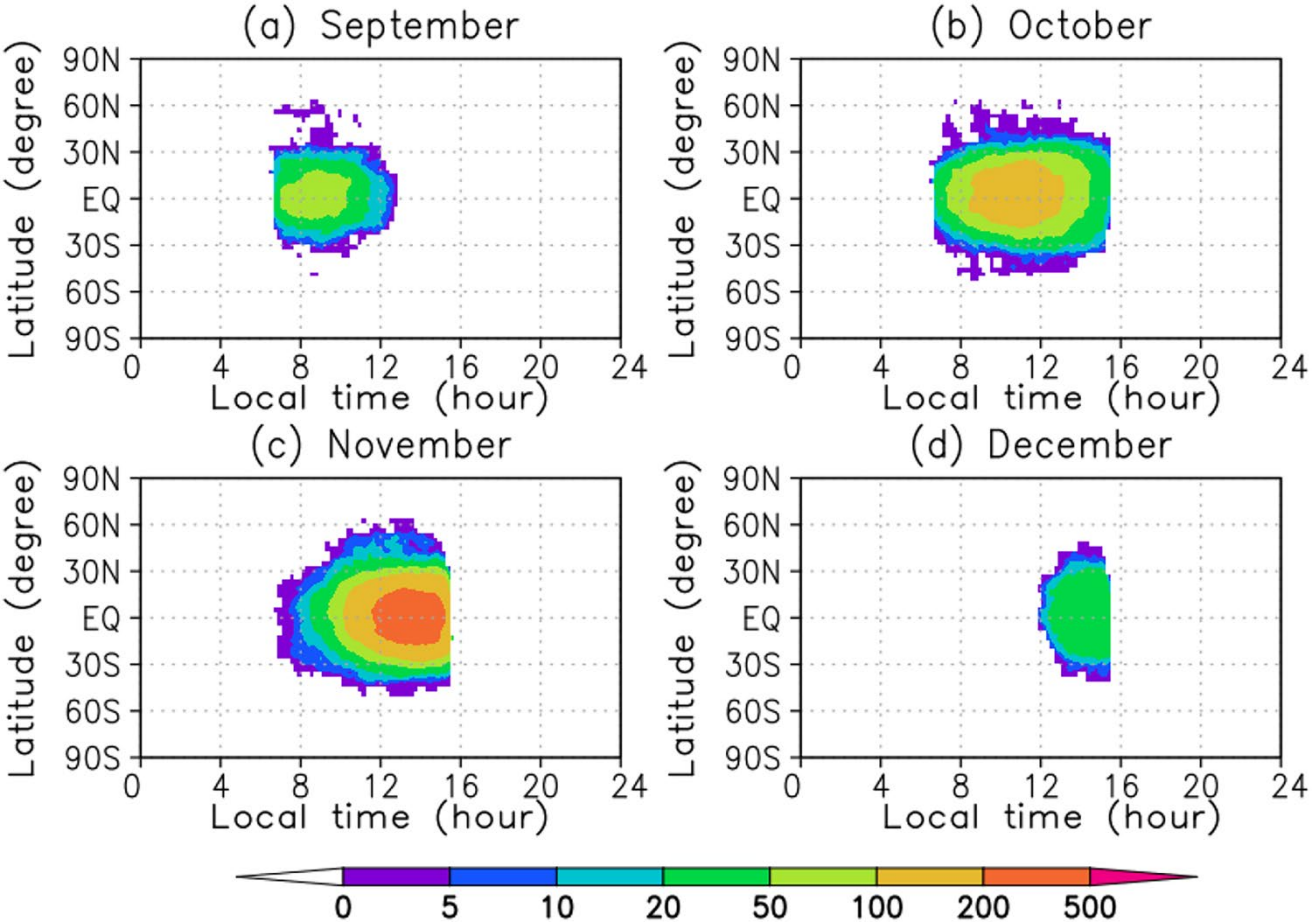
Local time and latitudinal distributions of the number of observations: (**a**) September, (**b**) October, (**c**) November and (**d**) December 2018.

The UVI image is obtained once every 2 h. There is no observation period from 26 to 27 November 2018 because the field of view of the camera is full of the nightside of Venus or no limb of Venus in the field of view for boresight correction associated with the pericenter passage. There are no observation data for some days from midnight to the morning for communication using a high gain antenna with the ground station on Earth. The number of observations per day is 6 and 13 at the lowest and highest, respectively (Fig. 5). The area with derived wind vectors is widest in November 2018, with data present from 7 to 15 LT (Fig. 6). In September 2018, most observations are in the morning, whereas they are in the afternoon in early December 2018. The spatial resolution is 120 × 60 horizontal grids, which is close to the resolution of AFES-Venus. It was assimilated at 70 km altitude, which is near the cloud top. The observation error was uniformly set to 4 m s^−1^ by simplifying the estimated latitude-dependent error profile^5^.

In this paper, only wind data obtained from UVI with 365 nm observations is assimilated. In the same observation period, there are wind data obtained from UVI with 283 nm observations and temperature data obtained from LIR observations. We will also proceed LIR temperature assimilation, though there are some difficulties to determine the sensing altitude. In the future, it will be possible to compare the assimilation of different types of observation data and to assimilate all these observation data simultaneously.

### Assessing the impact of observation on assimilation

To check if the assimilation is properly functioning, RMSD is calculated at a certain altitude as follows:

1 $$\begin{array}{*{20}c} {RMSD = \sqrt {\frac{1}{N}\mathop \sum \limits_{i = 1}^{N} \left( {X_{i} - x_{i} } \right)^{2} } } \\ \end{array} ,$$

where $X_{i}$ and $x_{i}$ are physical values in the FR and analysis for each case, respectively, and *N* is the total number of horizontal grid points. Thus, RMSD shows the difference between the FR and assimilation result; large RMSD indicates that the data assimilation significantly modified the model result.

**Figure 7:**
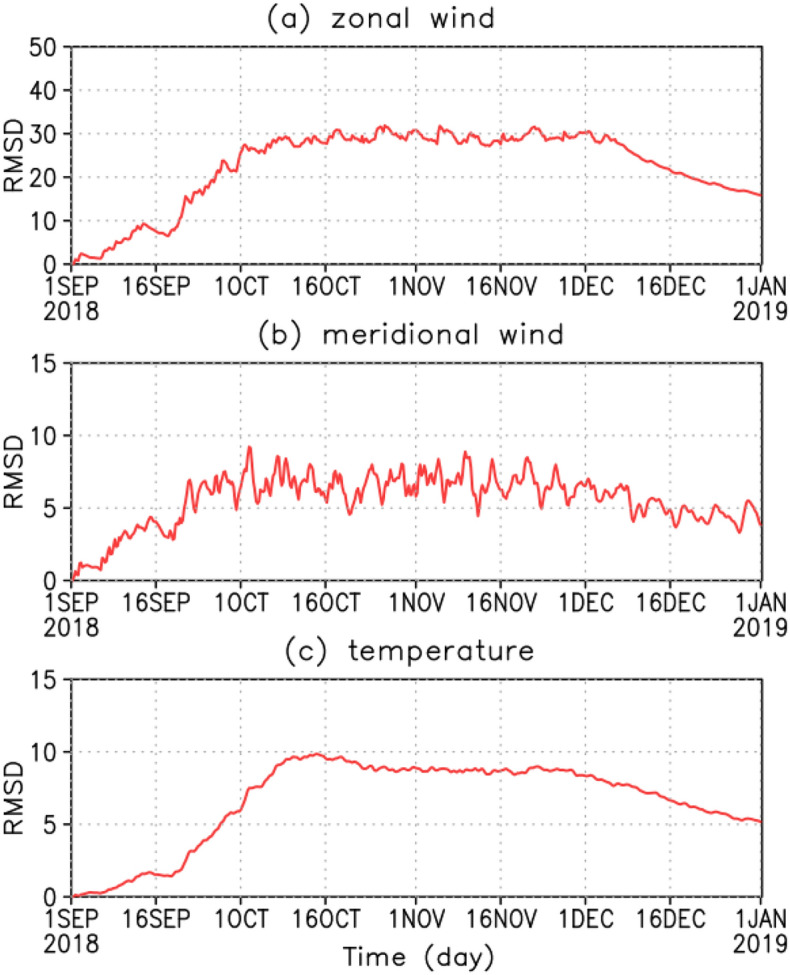
RMSD at 70 km altitude: (**a**) zonal wind, (**b**) meridional wind and (**c**) temperature.

The RMSD of zonal wind at 70 km altitude, where observations are assimilated, shows that the value increases after the end of September when observations become more frequent and gradually decreases in December after maintaining a constant value (Fig. 7a). Although not as prominent as of the zonal wind, the meridional wind has a similar tendency (Fig. 7b). If there are no observations to assimilate, the RMSD will converge to the natural ensemble spread of the model with time. On January 1, 2019, the final day of assimilation, the values were 18 and 4 m s^−1^ for the zonal and the meridional winds, respectively, which are not less than the observation error of 4 m s^−1^. Note that assimilation impacts can continue for more than 2 weeks. The temperature changes are similar to the zonal wind (Fig. 7c). Although only the horizontal wind was assimilated, the temperature also changed. In this study, we focus on the analysis during October–November 2018, when the RMSD values are stable and the observational data exist.

The data assimilation scheme derives improved estimates by combining observations with short-term forecast of each ensemble member with the numerical model. The results of short-term forecast are referred to as the background, and those assimilated with data are referred to as the analysis. Note that, in FR, the analysis and the background are equivalent. The ensemble spread is an index showing the magnitude of difference among the ensemble members of the background or the analysis, and is calculated by

2 $$\begin{array}{*{20}c} {SPRD = \sqrt {\frac{1}{M}\mathop \sum \limits_{j = 1}^{M} \left( {x_{j} - \overline{x}} \right)^{2} } ,} \\ \end{array}$$

where* M* is the number of ensemble members (in this study, *M* = 31). ${\varvec{x}}_{{\varvec{j}}} \left( {j = 1, \ldots , M} \right)$ is the state vector (e.g., zonal and meridional winds and temperature) of each ensemble member. $\overline{\user2{x}}$ is that of the ensemble mean. The ensemble increment is the difference between the ensemble mean for the background and the analysis and indicates the amount of correction by the data assimilation.

**Figure 8:**
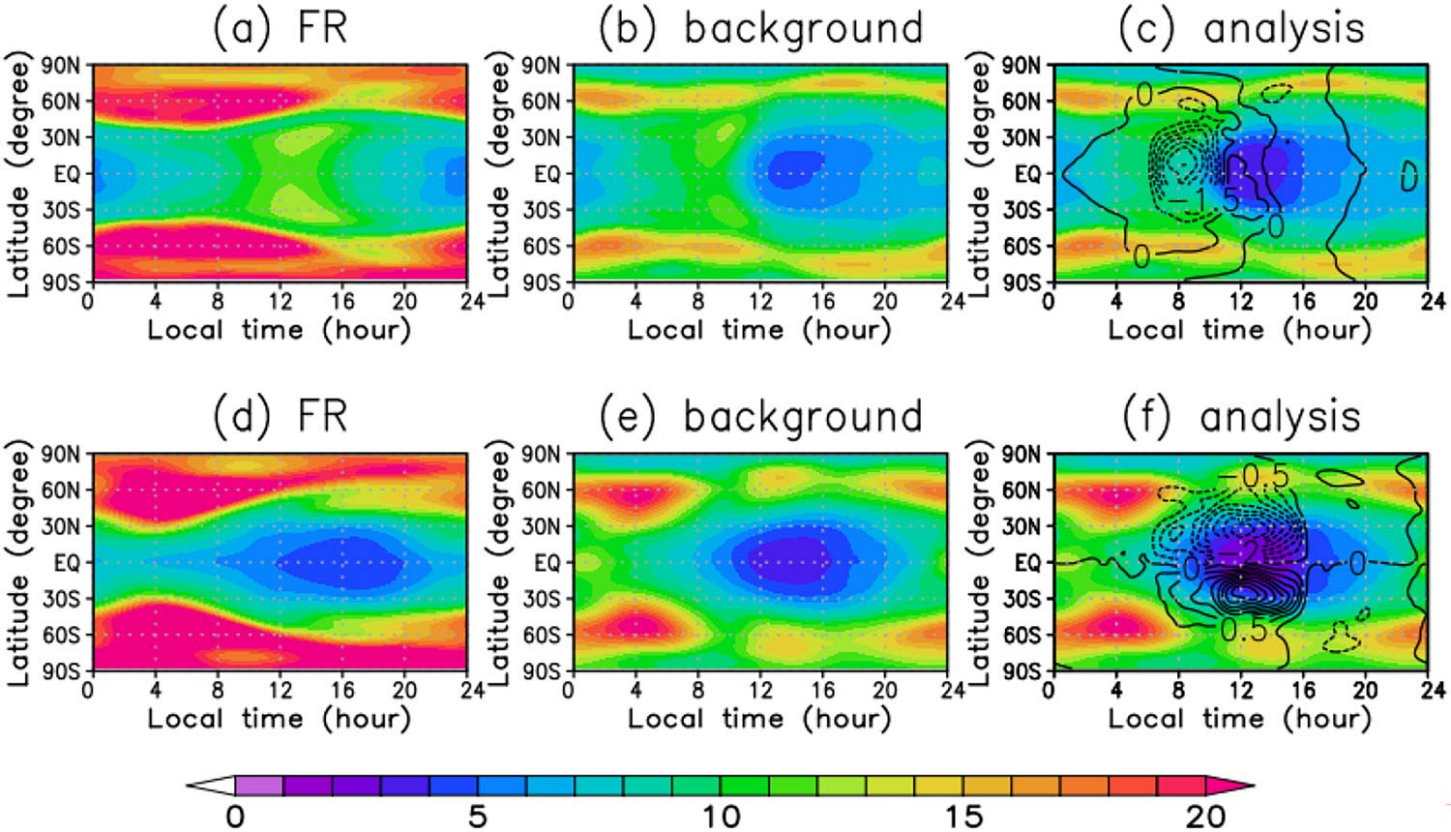
Local time and latitudinal distributions of ensemble spread (colour, m s^−1^) and ensemble increment (contour, m s^−1^) for (**a**, **b**, **c**) zonal winds and (**d**, **e**, **f**) meridional winds (m s^−1^) associated with thermal tides at the cloud-top (~ 70 km) level. (**a**, **d**) FR spreads, (**b**, **e**) background spreads and (**c**, **f**) analysis spreads and increments. The contour intervals of ensemble increment are 0.5 m s^−1^. These distributions are averaged for the two Earth months from 1 October to 30 November 2018 in a reference frame moving with the Sun to extract the structures associated with thermal tides.

Figure 8a–f show the ensemble spreads and the increments for the zonal and the meridional winds at the altitude of 70 km. They are averaged for the 2 months in a reference frame moving with the Sun. The FR spreads show that both zonal winds and meridional winds are larger than 20 m s^−1^ at mid- and high latitudes (Fig. 8a and d). The large magnitudes are considered to be due to the uncertainty of the baroclinic wave in the model. On the other hand, they are less than 15 m s^−1^ at low latitudes because the phases of the tidal waves are fairly fixed to the solar longitude for each ensemble member. Those results are related to the model bias in AFES-Venus that the phases of the thermal tides are different from observations as shown in Fig. 1a and b. The spreads of the background (Fig. 8b and e) show that both for the zonal winds and the meridional winds are globally smaller compared to those of FR, and the analysis spreads are particularly small compared to those of the background around noon in the equatorial region where the observations are assimilated (Fig. 8c and f). From these results, it can be assessed that the model bias can be corrected by the assimilation for both zonal winds and meridional winds, indicating that the model has a certain assimilation impact.

We have also calculated ensemble increment for zonal and meridional winds to see where the assimilation impact is high. The ensemble increment of the zonal wind has a local maximum of the absolute value at around 8 LT at the equatorial region for zonal winds, where the spread (around 10 m s^−1^) is larger than the observation error (4 m s^−1^) (Fig. 8c). The ensemble increment of the meridional wind has local maximum of the absolute value at around 30° N (S) latitude at noon, where the number of observations are large (Fig. 6b and c) and the spread is relatively large (around 5 m s^−1^) (Fig. 8f). At around 50° N (S) latitude, although the spread is large enough (around 10 m s^−1^), the observation frequency is low (Fig. 6b and c; less than 5 times in October and 10 times in November), which limits the amount of correction by the assimilation. The increments and spreads of the meridional wind can explain that the meridional wind is more than 20 m s^−1^ at 50° past noon, with only a slight difference between the analysis and FR (Fig. 1e and f), while the observation is around 10 m s^−1^, which is significantly different from the analysis (Fig. 1d and f).

### Estimation of angular momentum flux for tidal and other transient disturbances

The angular momentum flux in this study is estimated as follows. The direction of rotation in this model is the same as Earth (opposite to that of Venus), meaning that when the flux is positive, the angular momentum is transported northward. Note that the angular momentum flux results are opposite to the direction of UVI observations in Horinouchi et al.^5^. The total disturbance flux is defined as follows:

3 $$\begin{array}{*{20}c} {\langle u^{\prime}v^{\prime}\rangle_{total} = \left\{ {\overline{{\left( {u - \overline{u}} \right)\left( {v - \overline{v}} \right)}} } \right\}_{TA} } \\ \end{array}$$

where $u$ and $v$ are the zonal and meridional winds and are functions of latitude, LT, altitude and time, respectively; $\overline{\left( \cdot \right)}$ denotes the LT average, and $\left( \cdot \right)_{TA}$ denotes the time average. The angular momentum flux for thermal tides is defined as follows:

4 $$\begin{array}{*{20}c} {\langle u^{\prime}v^{\prime }\rangle_{tide} = \overline{{\left( {u_{TA} - \overline{{u_{TA} }} } \right)\left( {v_{TA} - \overline{{v_{TA} }} } \right)}} .} \\ \end{array}$$

Then, the fluxes of transient disturbances other than thermal tides are as given by

5 $$\begin{array}{*{20}c} {\langle {u^{\prime}v^{\prime}\rangle}_{transient disturbances} = u^{\prime}v^{\prime}_{total} - u^{\prime}v^{\prime}_{tide} } \\ \end{array}$$

## Data Availability

The datasets generated or analysed during this study are available from the corresponding author on reasonable request. The dataset of wind field acquired from the cloud tracking technique is available at https://doi.org/10.17597/isas.darts/vco-00020.

## Code availability

The model code of AFES-Venus is available from the corresponding author on reasonable request.

## References

[1] Nakamura M (2011). Overview of Venus orbiter, Akatsuki. Earth Planets Space.

[2] Nakamura M (2016). AKATSUKI returns to Venus. Earth Planets Space.

[3] Schubert G (1980). Structure and circulation of the Venus atmosphere. J. Geophys. Res..

[4] Horinouchi T (2018). Mean winds at the cloud top of Venus obtained from two-wavelength UV imaging by Akatsuki. Earth Planets Space.

[5] Horinouchi T (2020). How waves and turbulence maintain the super-rotation of Venus' atmosphere. Science.

[6] Rossow WB (1980). Cloud morphology and motions from pioneer Venus images. J. Geophys. Res..

[7] Kashimura H (2019). Planetary-scale streak structure reproduced in a Venus atmospheric simulation. Nat. Commun..

[8] Titov DV (2012). Morphology of the cloud tops as observed by the Venus Express Monitoring Camera. Icarus.

[9] Fukuhara T (2017). Large stationary gravity wave in the atmosphere of Venus. Nat. Geosci..

[10] Ingersoll AP, Orton GS (1974). Lateral inhomogeneities in the Venus atmosphere: Analysis of thermal infrared maps. Icarus.

[11] Schofield JT, Taylor FW (1983). Measurements of the mean, solar-fixed temperature and cloud structure of the middle atmosphere of Venus. Q. J. R. Meteor. Soc..

[12] Limaye SS (1988). Venus: Cloud level circulation during 1982 as determined from pioneer cloud photopolarimeter images: II Solar longitude dependent circulation. Icarus.

[13] Zasova LV (2007). Structure of the Venus atmosphere. Planet. Space Sci..

[14] Kouyama T (2019). Global structure of thermal tides in the upper cloud layer of Venus revealed by LIR on board Akatsuki. Geophys. Res. Lett..

[15] Del Genio AD, Rossow WB (1990). Planetary-scale waves and the cyclic nature of cloud top dynamics on Venus. J. Atmos. Sci..

[16] Imai M (2019). Planetary-scale variations in winds and UV brightness at the venusian cloud top: Periodicity and temporal evolution. J. Geophys. Res. Planets.

[17] Nara Y (2019). Formation of the Y feature at the venusian cloud top by planetary-scale waves and the mean circulation: Analysis of Venus Express VMC images. J. Geophys. Res..

[18] Kajiwara N (2021). Planetary-scale waves seen in thermal infrared images of venusian cloud top. J. Geophys. Res. Planets.

[19] Ingersoll AP (1987). Estimates of convective heat fluxes and gravity wave amplitudes in the Venus middle cloud layer from VEGA balloon measurements. Adv. Space Res..

[20] Peralta J (2008). Characterization of mesoscale gravity waves in the upper and lower clouds of Venus from VEX-VIRTIS images. J. Geophys. Res..

[21] Piccialli A (2014). High latitude gravity waves at the Venus cloud tops as observed by the Venus Monitoring Camera on board Venus express. Icarus.

[22] Ando H (2015). Vertical wavenumber spectra of gravity waves in the Venus atmosphere obtained from Venus express radio occultation data: Evidence for saturation. J. Atmos. Sci..

[23] Imamura T (2018). Fine vertical structures at the cloud heights of Venus revealed by radio holographic analysis of Venus express and Akatsuki radio occultation data. J. Geophys. Res..

[24] Mori R (2021). Gravity wave packets in the venusian atmosphere observed by radio occultation experiments: Comparison with saturation theory. J. Geophys. Res. Planets.

[25] Sánchez-Lavega A (2017). The atmospheric dynamics of Venus. Space Sci. Rev..

[26] Kalnay E (1996). The NCEP/NCAR 40-year reanalysis project. Bull. Am. Meteor. Soc..

[27] Montabone L (2014). The Mars analysis correction data assimilation (MACDA) dataset V1.0. Geosci. Data J..

[28] Miyoshi T, Yamane S (2007). Local ensemble transform Kalman filtering with an AGCM at a T159/L48 resolution. Mon. Weather Rev..

[29] Miyoshi T (2007). The AFES-LETKF experimental ensemble reanalysis: ALERA. SOLA.

[30] Sugimoto N (2014). Baroclinic instability in the Venus atmosphere simulated by GCM. J. Geophys. Res. Planets.

[31] Sugimoto N (2017). Development of an ensemble Kalman filter data assimilation system for the venusian atmosphere. Sci. Rep..

[32] Sugimoto N (2019). Impact of data assimilation on thermal tides in the case of Venus Express wind observation. Geophys. Res. Lett..

[33] Tomasko MG (1980). Measurement of the flux of sunlight in the atmosphere of Venus. J. Geophys. Res..

[34] Fels SB, Lindzen RS (1974). The interaction of thermally excited gravity waves with mean flows. Geophys. Fluid Dyn..

[35] Pechmann JB, Ingersoll AP (1984). Thermal tides in the atmosphere of Venus: Comparison of model results with observations. J. Atmos. Sci..

[36] Baker NL, Leovy CB (1987). Zonal winds near Venus’ cloud top level: A model study of the interaction between the zonal mean circulation and the semidiurnal tide. Icarus.

[37] Newman M, Leovy C (1992). Maintenance of strong rotational winds in venus’ middle atmosphere by thermal tides. Science.

[38] Takagi M, Matsuda Y (2007). Effects of thermal tides on the Venus atmospheric superrotation. J. Geophys. Res..

[39] Lebonnois S (2010). Superrotation of Venus’ atmosphere analyzed with a full general circulation model. J. Geophys. Res..

[40] Yamazaki A (2018). Ultraviolet imager on Venus orbiter Akatsuki and its initial results. Earth Planets Space.

[41] Ando H (2018). Local time dependence of the thermal structure in the venusian equatorial upper atmosphere: Comparison of Akatsuki radio occultation measurements and GCM results. J. Geophys. Res. Planets.

[42] Horinouchi T (2021). Venus climate orbiter Akatsuki cloud motion vector data set v1.0. JAXA Data Arch. Transm. Syst..

[43] Rossow WB (1990). Cloud-tracked winds from pioneer Venus OCPP images. J. Atmos. Sci..

[44] Khatuntsev IV (2013). Cloud level winds from the Venus express monitoring camera imaging. Icarus.

[45] Hueso R (2015). Six years of Venus winds at the upper cloud level from UV, visible and near infrared observations from VIRTIS on Venus Express. Planet. Space Sci..

[46] Takagi M (2018). Three dimensional structures of thermal tides simulated by a Venus GCM. J. Geophys. Res. Planets.

[47] Fukuya K (2021). The nightside cloud-top circulation of the atmosphere of Venus. Nature.

[48] Taguchi M (2007). Longwave infrared camera onboard the Venus climate orbiter. Adv. Space Res..

[49] Sugimoto N (2019). Observing system simulation experiment for radio occultation measurements of the Venus atmosphere among small satellites. J. Jpn. Soc. Civ. Eng. A2 Appl. Mech..

[50] Sugimoto N (2021). Observing system simulation experiment to reproduce Kelvin wave in the Venus atmosphere. Atmosphere.

[51] Sugimoto N (2022). Kelvin wave and its impact on the Venus atmosphere tested by observing system simulation experiment. Atmosphere.

[52] Kouyama T (2015). Vertical propagation of planetary-scale waves in variable background winds in the upper cloud region of Venus. Icarus.

[53] Crisp D (1986). Radiative forcing of the Venus mesosphere I Solar fluxes and heating rates. Icarus..

[54] Seiff A (1985). Models of the structure of the atmosphere of Venus from the surface to 100 kilometers altitude. Adv. Space Res..

[55] Sugimoto N (2014). Waves in a Venus general circulation model. Geophys. Res. Lett..

[56] Ikegawa S, Horinouchi T (2016). Improved automatic estimation of winds at the cloud top of Venus using superposition of cross-correlation surfaces. Icarus.

[57] Horinouchi T (2017). Image velocimetry for clouds with relaxation labeling based on deformation consistency. Meas. Sci. Technol..

